# Mathematical Model of Neuronal Morphology: Prenatal Development of the Human Dentate Nucleus

**DOI:** 10.1155/2014/812351

**Published:** 2014-06-05

**Authors:** Katarina Rajković, Goran Bačić, Dušan Ristanović, Nebojša T. Milošević

**Affiliations:** ^1^Laboratory for Image Analysis, Medical Faculty, University of Belgrade, Serbia; ^2^Faculty of Physical Chemistry, University of Belgrade, Serbia; ^3^Department of Biophysics, Medical Faculty, University of Belgrade, KCS Institute of Biophysics PP. 22, 11129 Belgrade, Serbia

## Abstract

The aim of the study was to quantify the morphological changes of the human dentate nucleus during prenatal development using mathematical models that take into account main morphometric parameters. The camera lucida drawings of Golgi impregnated neurons taken from human fetuses of gestational ages ranging from 14 to 41 weeks were analyzed. Four morphometric parameters, the size of the neuron, the dendritic complexity, maximum dendritic density, and the position of maximum density, were obtained using the modified Scholl method and fractal analysis. Their increase during the entire prenatal development can be adequately fitted with a simple exponential. The three parameters describing the evolution of branching complexity of the dendritic arbor positively correlated with the increase of the size of neurons, but with different rate constants, showing that the complex development of the dendritic arbor is complete during the prenatal period. The findings of the present study are in accordance with previous crude qualitative data on prenatal development of the human dentate nucleus, but provide much greater amount of fine details. The mathematical model developed here provides a sound foundation enabling further studies on natal development or analyzing neurological disorders during prenatal development.

## 1. Introduction

It seems that many disorders of the cerebellum, such as cerebellar atrophy, ataxia, dysdiadochokinesia, and intention tremor, may be developmental in origin [1]. To recognize impaired development and understand the etiology of various neurological pathological disorders of the cerebellum, a precise timetable of the cellular events that take place during normal development is needed [2]. Some data are available on the neuronal types of the dentate nucleus in rats [3], monkeys [3–5], cats [6], and adult humans [7].

However, despite the critical role of the dentate nucleus in the cerebellar function and motor control, only a few studies have dealt with prenatal development of this deep cerebellar nucleus in humans [8–12]. During the period from 8 gestational weeks to 10 years, the human dentate nucleus changes in size and shape, as well as in its neuronal composition [1]. Detailed morphological analysis of Golgi and Nissl staining of the neonatal human dentate nucleus documented the presence of various cell types and their development [8–10, 12]. Although the growth of dendrites and the formation of a characteristic dendritic arbor constitute one of the major morphogenetic events in the prenatal development of a neuron, these aspects were not adequately analyzed in those studies. Authors attempted to provide parameters (the dendritic field and the number of dendritic intersections) of neuronal morphology during gestation by using rather crude measurements of the overall size of the dendritic field and loosely defined parameters describing criteria of branching complexity [12]. Consequently, a detailed quantification of branching complexity of the dendritic arbor neurons of the dentate nucleus during its development in the prenatal period is lacking.

Thus, this study attempts to quantify the morphology of neurons from the dentate nucleus at various gestational periods, analyzing the size of neurons and branching complexity of dendritic arbor. Dendritic branching were investigated using models developed in some previous studies for the adult dentate nucleus [13, 14]. While the global fractal dimension of a neuronal dendritic arbor has been suggested as a useful quantifier measuring the degree of dendrite aberrations from straight lines and completeness of filling the dendritic field with dendrites [15–17], the modification of the Sholl method estimates the place of a possible circle intersecting maximum number of dendrites and the maximum number of intersections measures the maximum density of dendritic arbor [14]. These parameters can exactly quantify dendritic arbor for each reconstructed neuron. In addition, the correlation between the size and complexity of neurons was investigated by mathematical and statistical methods and the model of morphological changes during prenatal development is proposed.

## 2. Materials and Methods

### 2.1. Image Gathering and Acquisition

The camera lucida drawings of Golgi impregnated neurons, taken from human fetuses of gestational ages ranging from 14 to 41 weeks, were taken from [8–10, 12] with permission. Details on fetal cadavers, histological procedure, fetal data, and original images can be found therein. Briefly, the gestational age of the fetuses has been estimated by taking into consideration the fetal crown rump length, biparietal diameter, the foot length, and the maternal history of the last menstrual period and the size of the uterus [9, 12]. Included in this study were only fetuses where no external abnormalities of the brain or any disease of the central nervous system were detected [2].

**Figure 1:**
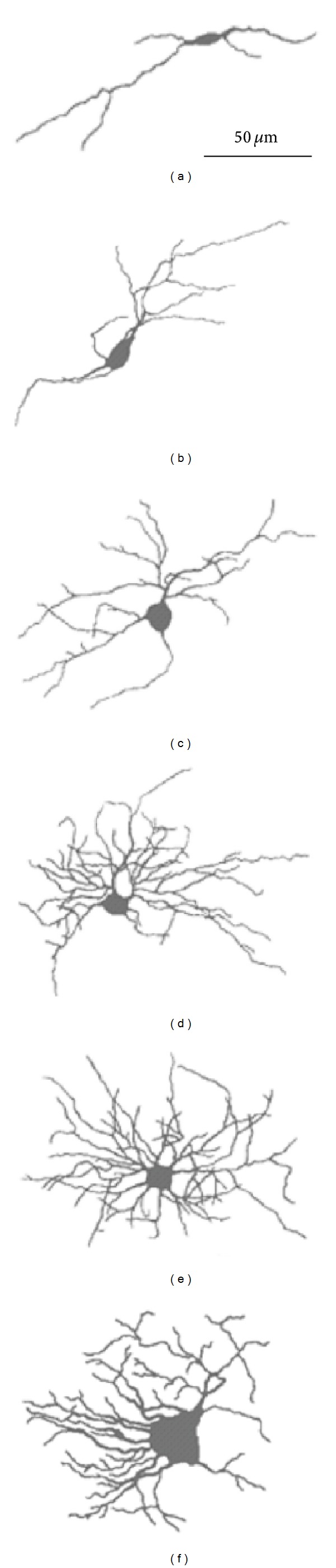
Representative examples of large neurons of the human dentate nucleus during prenatal development: (a) 14.5 gw, (b) 19.5 gw, (c) 24 gw, (d) 27.5 gw, (e) 34 gw and (f) 41 gw. The original images can be found in [8–10, 12].

The drawings were scanned into the PC computer at the highest available resolution (1200 dpi) in order to obtain two-dimensional (2D) digitized images. According to previous criteria and image quality, a total of 81 images was selected and roughly divided into the following: 11 images were from 14 to 15 gestational weeks (gw), 13 images from 19 to 20 gw, 11 images from 24 gw, 16 images from 27 to 28 gw, 15 images from 34 gw and 15 images from 41 gw.  Figure 1 shows representative examples of such neurons at different gestational times.

### 2.2. Image Analysis

All digitized images were imported into Image J, specialized public domain software for image analysis, developed by the National Institutes of Health (USA, http://rsb.info.nih.gov/ij). The neuronal morphology was quantitatively evaluated with four parameters which describe two cell features: neuron size and branching complexity of the dendritic arbor.

The size of neurons was estimated by measuring the neuron area (*A*) in accordance with the procedure outlined earlier [18]. Briefly, the binary image of neuron was imported in Image J and number of pixels was calculated (Analyze : Histogram : List). The neuron area was estimated after number of pixels of the skeletonized scale bar (50 *μ*m) was obtained and the distance of one pixel was converted in *μ*m^2^.

**Figure 2:**
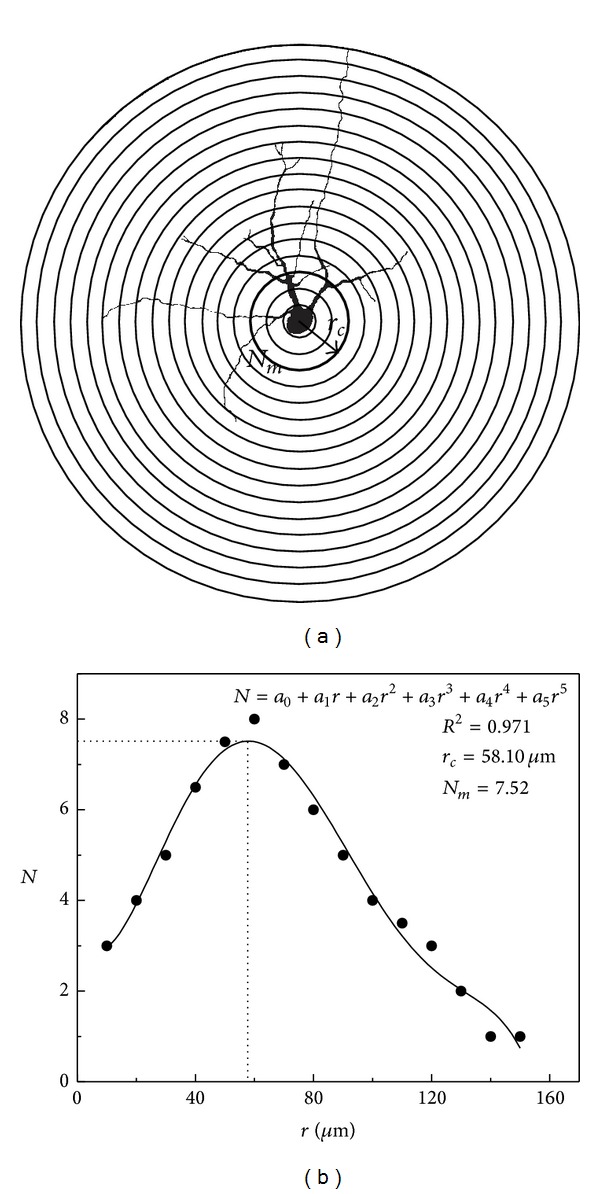
Illustration of an application of the modified Sholl method to a dentate nucleus neuron: (a) numbers of intersections of (*N*) versus radius dimension (*r*) were fitted by the polynomial of the fifth order and their values are shown in (b). *R* ^2^ is the coefficient of determination of the fit, while the values *N*_*m*_ and *r*_*c*_ were calculated according to [14].

The dendritic branching complexity was investigated after the 2D images of the neurons were skeletonized and subjected to box-count [15] and modified Sholl analysis [14]. By means of these quantitative techniques, three parameters were obtained: the global fractal dimension (*D*), maximum number of intersections (*N*_*m*_), and critical radius (*r*_*c*_). The *D* was obtained by the box-counting method: a detail procedure was reported in [13, 15]. Two parameters of the modified Sholl analysis were calculated when the image of neurons was overlaid with a series of equidistantly arranged concentric circles centered in the cell body (Figure 2(a)). The scatter diagram between numbers of intersections of dendrites (*N*) with these circles and circle radii (*r*) were fitted by a polynomial of the fifth degree [14], and the critical radius *r*_*c*_ and maximum *N*_*m*_ of *N*(*r*) were estimated (Figure 2(b)).

### 2.3. Statistical Analysis

Statistical analysis of the calculated morphometric parameters depends on whether the distribution is normal or not [19]. The number of neurons in each group of a certain gestational age was relatively small, that is, smaller than 30 (see Section 2.1); hence, the testing of the distribution character has to be based on the calculation of two statistical parameters: skewness (*a*_3_) and excess of distribution (*e*) [20]. In brief, the intervals of skewness and excess values are estimated when these two parameters are divided by the corresponding mean square errors (*σ*_3_ and *σ*_4_). If the absolute value of the quotients *σ*_3_/*a*_3_ and *σ*_4_/*e* is less than or equal to 2, then the data distribution can be considered as normal [20].

With the aim of finding a function which best models the changes of all obtained morphometric parameters we fitted each set of data with a variety of functions. The selection criteria for the tested function were as follows: to have a simple structure, to fit the data instead of interpolating them, and to have the correlation coefficient higher than 0.85. Final statistical evaluation of the function which fits the experimental data is based on the coefficient of determination (*R* ^2^) and standard error (SE) of the fitted function [21]. These parameters are defined by the following equations:

(1) $$\begin{array}{c} R^{2}=1-\overset{N}{\underset{1}{\sum }}\frac{{\left(y_{i}-f_{i}\right)}^{2}}{{\left(y_{i}-y_{\text{av}}\right)}^{2}},\\ \text{SE}=\sqrt{\frac{\sum_{1}^{N}{\left(y_{i}-f_{i}\right)}^{2}}{N\left(N-1\right)},} \end{array}$$

where *y*_*i*_ are experimental data, *f*_*i*_ are fitted data, *y*_av_ is the average of experimental data, and *N* is the number of data points [21].

Statistical significance between means of morphometric parameters in a certain gestational age was estimated by the Scheffé post hoc test, while the correlation between some morphometric parameters was performed using the Spearman-rank correlation tests [21]. The chosen fit (i.e., the mathematical model of the growth) was evaluated by one-way ANOVA.

## 3. Results

### 3.1. Cell Morphology during Gestation

Table 1 shows values of *a*_3_, *e*, *σ*_3_, and *σ*_4_ for all four morphometric parameters at six gestational times. The absolute ratios (*σ*_3_/*a*_3_) and (*σ*_4_/*e*) are smaller than the critical value of 2 in all cases; thus, the calculated values of all parameters are distributed normally. Consequently, their values can be expressed by the mean value and standards error (Table 2).

Table 2 shows means and standard errors for *A*, *D*, *N*_*m*_, and *r*_*c*_ for six times of the gestation. It looks obvious that there is a concomitant increase of both the neuron area and their complexity during growth. To prove this assumption, we statistically analyzed each of the four morphometric parameters between consecutive weeks of gestation (see end of Section 2.3) and calculated *t* values were shown in Table 3. Symbol ∗ denotes significant difference from *P* < 0.05 to *P* < 0.001.

All parameters increased between 14.5 and 24 gw, except the mean *r*_*c*_ which remained statistically the same between 19.5 and 24 gw. The mean *D* and *N*_*m*_ increased slowly but significantly between 24 and 27.5 gw. Starting from 27.5 gw, there were no significant changes in any parameters, except a statistically significant increase in *A* up to 41 gw.

### 3.2. The Growth of Neurons

As we stated before, among many functions, which fulfill previous conditions (Section 2.3), three functions emerged: (i) linear, (ii) power, and (iii) exponential, defined by

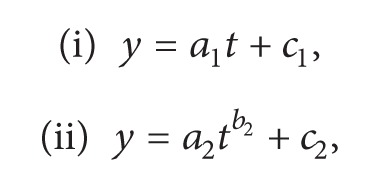
(2)

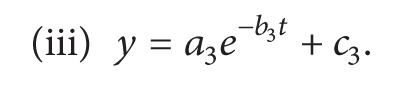
(3)


**Figure 3:**
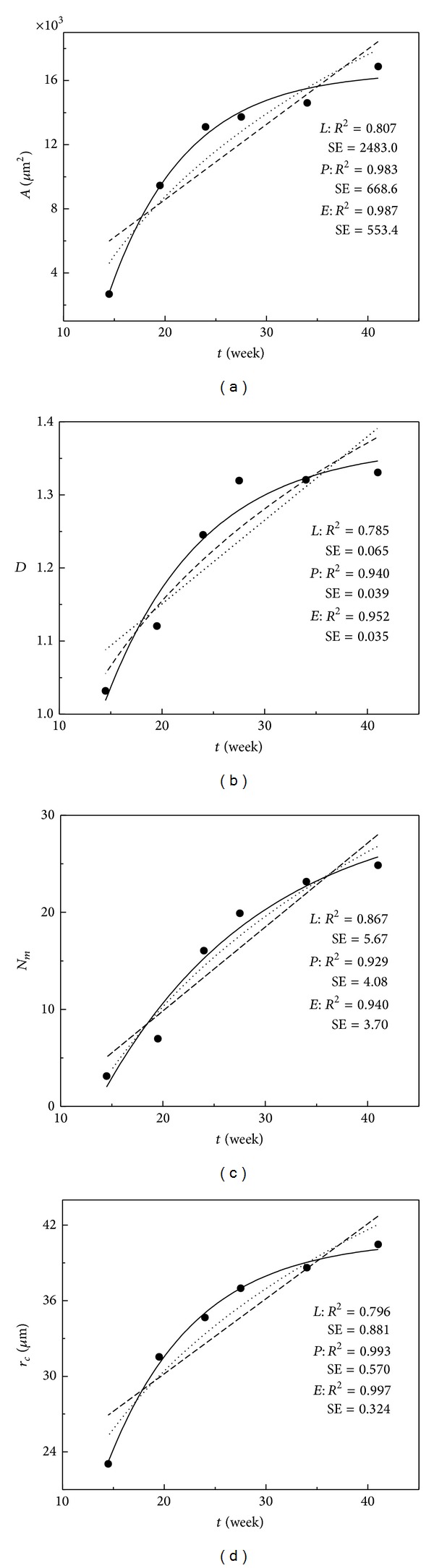
The linear (*L*-dotted line), power (*P*-dashed line), and exponential (*E*-solid line) function fitting of the data of increase of the neuron area (a), fractal dimensions (b), maximum number of intersections (c), and critical radius (d) during gestation. *R* ^2^ and SE for each fit are inserted in the plot.

Figure 3 shows the plot of each function for the mean *A*, *D*, *N*_*m*_, and *r*_*c*_ during gestation. It is obvious that the exponential function of the highest values of *R* ^2^ and the smallest values of SE is the best solution for fitting the changes of all four parameters during gestation (see Section 2.3).

The model of the growth, showed by (3), was further examined by one-way ANOVA (Table 4). The Fisher statistic (*F* value), for all parameters, was higher than the critical value (*F*_*t*_ = 28.2) at *P* = 0.05 the level of significance. This result indicated that the model was significant at a high confidence level (more than 95%, see Table 4).

The values for coefficients *a*_3_, *b*_3_, and *c*_3_ (3), for each of the four morphometric parameters, are presented in Table 5. It is obvious that values of all parameters tend to saturate during gestation. The parameter *c*_3_ represents the horizontal asymptote of the function or the maximum value of morphometric parameters when time converges to infinity. The parameter *b*_3_ (week^−1^) is the rate constant of exponential increase. The rate constant for *A* has a higher value than for all other three parameters (Table 5).

### 3.3. The Size of Neurons versus Branching Complexity during Gestation

It is obvious from previous data that it is important to analyze how different parameters of the branching complexity are correlated to the size of neurons. To explore whether there were statistical significance between three pairs of parameters (dendritic branching complexity versus size of the neuron), a one-way ANOVA was used (Table 4). According to results, the influence between *D*, *N*_*m*_, and *r*_*c*_ on *A* were statistically significant at a 95% confidence level.

**Figure 4:**
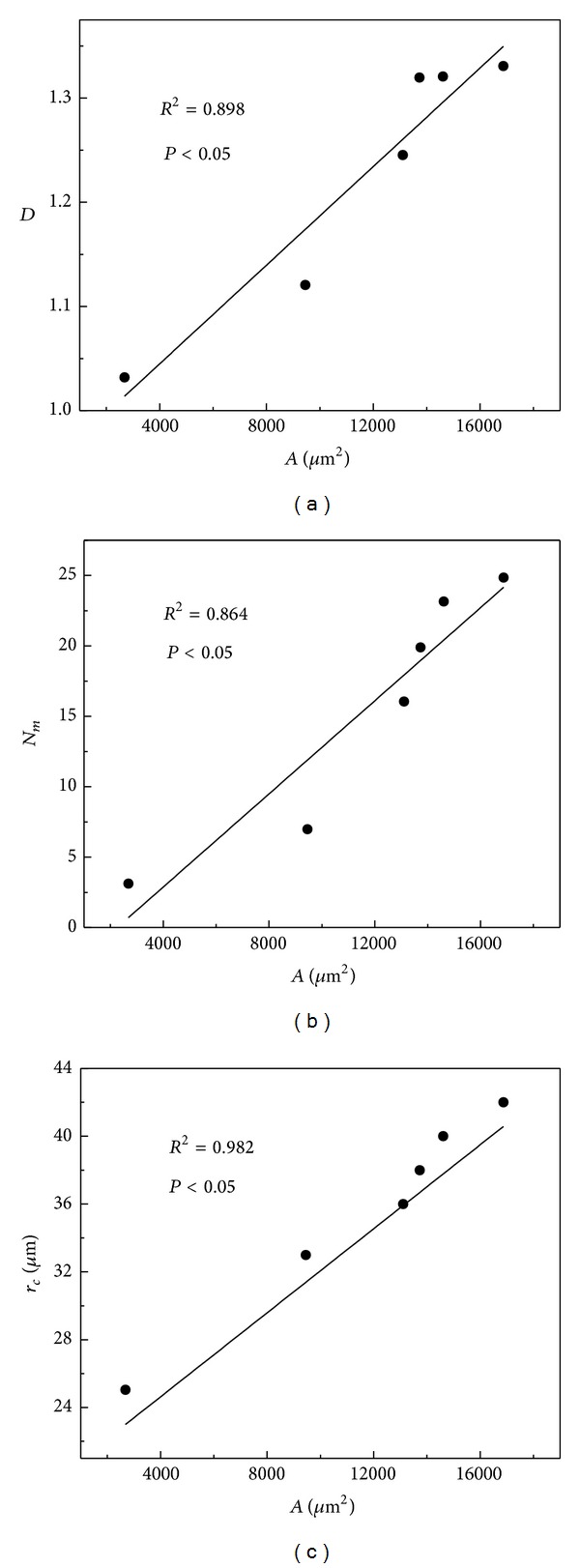
(a)–(c): Linear correlation between the mean area (*A*) and three parameters of dendritic arbor: the mean fractal dimension *D*, the mean maximum number of intersections *N*_*m*_, and the mean critical radius *r*_*c*_.

To resolve how the dendritic tree was altered with the increase in neuron size, a plot between each parameter of dendritic complexity and the size of the neuron was investigated (Figure 4). Using the Spearman-Rank correlation, a positive linear correlation was obtained between *D* and *A*, *N*_*m*_, and *A*, as well as between *r*_*c*_ and *A* (see confidence level in Figure 4).

## 4. Discussion

The dentate nucleus occupies a central position in the cerebellar circuitry, serving as a relay center for fibers coming from the cerebellar cortex, namely, from the axons of Purkinje cells [3]. It is noteworthy that the human dentate nucleus has a protracted developmental period extending over seven to eight months of intrauterine life thereby rendering it vulnerable to environmental hazards and stimuli [1, 11]. Illustrations in Figure 1 clearly show that it is impossible to analyze the morphology of neurons during development, particularly the branching complexity, without using some mathematical analyses. Thus, to our knowledge, this is the first time that a mathematical model which quantitatively describes the neuron size and branching complexity of the dendritic arbor in prenatal development is used.

Simultaneously using two mathematical analyses (Section 2.2), we calculated four parameters which quantify two key cell features: the size of the neuron field and branching complexity of the dendritic arbor. Similar analyses have been performed previously [7, 18], but these were aimed at classifying fully developed large neurons in adult humans. On the other hand, attempts to quantify development of the human dentate nucleus during gestation [9, 12] based on rather crude methods resulted in an arbitrary division into 3-4 developmental periods. It is unlikely that some sharp distinctions between neurons exist during gestation (i.e., discontinuities).

Our results suggest that changes of all morphometric parameters during the gestational age can be presented with a simple exponential function whose parameters can be reasonably related to morphological features of neurons. It is plausible that a better fit can be obtained by some other function (e.g., high order polynomial), but it is questionable how an increased number of fitting parameters can be connected to actual morphological features.

Previous qualitative analyses were based on the morphometric analysis of discrete types of neurons which appear during the development of human dentate neurons (e.g., bipolar versus multipolar, hemispheric versus pear shaped, etc.) and accordingly development has been divided into discrete time periods [8, 9, 12, 22]. Semiquantitative measurements were performed by measuring the mean body diameter and dendritic field diameter, but given the development stage of computers at that time, it was virtually impossible to quantitatively correlate these parameters with the branching complexity of the dendritic arbor as seen in morphometry and/or to analyze interconnections of different aspects of branching. Here we present a rather complex analysis where four growth parameters were determined and the relationship between them can be easily established.

Using more than one parameter to describe dendritic complexity allows more subtle analysis of changes during prenatal growth than was possible in previous works. For example, Hayaran et al. [12] have found an intense increase in the complexity of neurons during weeks 15 and 24 gw and a slow increase up to 28 gw. No further changes were observed up to 34 gw. Similarly, Mihajlovic and Zecevic [9] have found a profuse increase of branching complexity up to 25 gw followed by a slow maturation process. Analysis of changes of morphometric parameters during gestation presented here essentially confirms previous qualitative analysis. However, the pattern for four parameters is different. All parameters increase during gestation but with different rate constants (see *b*_3_ values in Table 5). Also, the increase of critical radius saturates at 19.5 gw values, while values of the global fractal dimension and maximum number of intersections continue to increase up to 27.5 gw and values of the neuron area increase during the entire examined period. It is premature and beyond the scope of this publication to speculate on the physiological significance of such analysis, but it should be noted that this study was performed on a large number of samples (81 images in different stages), while previous studies [8–10, 12] have been performed on a much smaller number of specimens.

Our results also suggest that the development of the dendritic arbor, as described by three morphologic parameters, is essentially completed during the prenatal period which is in agreement with work of Hayaran et al. [12], where also one type of Scholl analysis has been performed. The other study [9], based on mostly qualitative criteria, also confirms that the major developments of dendritic branching are completed in the prenatal stage and also that some slow and subtle maturing consisting of secondary and tertiary branches continues into the postnatal period. The major problem is that there are virtually no studies where the status of the dendritic arbor of prenatal and adults is compared using the same methodology.

We are in a unique position to accomplish that since dendritic branching patterns in adults have been analyzed using the same models in our previous publications [7, 18]. When values of global fractal dimension, maximum number of intersections, and critical radius for prenatal at 41 gw (Table 2) are compared with those of adults [7] the following emerges: (i) the global fractal dimension is essentially the same (1.32 versus 1.30); (ii) the critical radius is also essentially the same (42 *μ*m versus 43). Interestingly the critical radius value of 40 *μ*m for 28 gw has been found in another study which used a crude Scholl analysis [12]; (iii) surprisingly the maximal number of intersections is higher in prenatal than in adults (25 versus 16), which can be explained by transient side branching during the prenatal stage. Similar features were observed in cats where delicate structuring of dendrites occurs in prenatal and neonatal but is absent in adults [23]. All that suggests that the major development of the dendritic arbor is completed during the prenatal phase, where it reaches almost its final form and that fine remodeling can occur in the natal period.

## 5. Conclusion

In conclusion, this is the first study where development of the dentate nucleus during gestation in humans was investigated using quantitative analysis of morphology. Three morphologic parameters obtained from both Scholl and fractal analysis (the global fractal dimension of the arbor, the critical radius of the arbor, and the maximal number of intersections of dendrites) were correlated with the changes of neuron size. All parameters were positively correlated with the increase of the size of neurons, but with different increase rate constants. All results show that the development of the dendritic arbor is almost completed during the prenatal period. This study provides a good platform for further studies of quantification of morphology of dentate neurons throughout postnatal development or in cases where some neurological disorder occurred.

## Figures and Tables

**Table 1:** The values of skewness (*a*_3_), excess (*e*), and corresponding standard errors (*σ*_3_ and *σ*_4_) for the neuron area (*A*), fractal dimension (*D*), maximum number of intersections (*N*_*m*_), and critical radius (*r*_*c*_) at the six gestational times.

Morphometric parameters	*t* (week)	*a*_3_	*e*	*σ*_3_	*σ*_4_
*A*	14.5	0.55	−0.88	0.63	0.95
	19.5	−0.34	−1.03	0.59	1.00
	24	−1.84	0.89	0.56	0.89
	27.5	0.55	0.94		
	34	−1.13	−0.92	0.55	0.88
	41	−0.50	−1.03	0.63	0.95

*D*	14.5	0.61	−0.76	0.63	0.95
	19.5	−0.33	−0.90	0.59	1.00
	24	−0.31	−1.75	0.56	0.89
	27.5	0.57	1.14		
	34	0.34	−0.87	0.55	0.88
	41	−0.35	−1.22	0.63	0.95

*N*_*m*_	14.5	0.40	1.00	0.63	0.95
	19.5	0.82	−0.65	0.59	1.00
	24	0.73	3.85	0.56	0.89
	27.5	1.43	13.01		
	34	1.36	1.13	0.55	0.88
	41	−1.14	1.41	0.63	0.95

*r*_*c*_	14.5	−0.49	0.67	0.63	0.95
	19.5	−0.36	3.13	0.59	1.00
	24	0.37	3.42	0.56	0.89
	27.5	0.59	10.05		
	34	0.54	1.18	0.55	0.88
	41	−0.35	1.94	0.63	0.95

**Table 2:** The values of the neuron area (*A*), fractal dimension (*D*), maximum number of intersections (*N*_*m*_), and critical radius (*r*_*c*_) for the six gestational times.

*t* (week)	*A* (*μ*m^2^)	*D*	*N*_*m*_	*r*_*c*_ (*μ*m)
14.5	2700 ± 300	1.034 ± 0.007	3.0 ± 0.2	25 ± 1
19.5	9500 ± 700	1.125 ± 0.008	7.9 ± 0.5	33 ± 3
24	13100 ± 600	1.24 ± 0.01	16 ± 2	36 ± 3
27.5	13700 ± 500	1.313 ± 0.007	23 ± 2	38 ± 1
34	14600 ± 500	1.319 ± 0.006	24 ± 1	40 ± 2
41	16900 ± 700	1.326 ± 0.006	25 ± 1	42 ± 4

Each value is presented as the mean ± SE.

**Table 3:** Calculated *t* values estimated the significance for four morphometric parameters between five successive pairs of gestational ages.

Pairs	Calculated *t* values			
	*A* (*μ*m^2^)	*D*	*N*_*m*_	*r*_*c*_ (*μ*m)
14.5-19.5	7.731***	8.228***	8.152***	2.095*
19.5-24	6.093***	8.695***	4.945***	0.774
24-27.5	0.732	6.671***	2.590*	0.478
27.5-34	1.097	0.661	0.484	0.939
34-41	2.729*	0.814	0.465	0.515

Significant differences are denoted with the symbol: *P < 0.05 and ***P < 0.001.

**Table 4:** The results of one-way ANOVA applied to the fit (presented with (3)) for the neuron area (*A*), fractal dimension (*D*), maximum number of intersections (*N*_*m*_), critical radius (*r*_*c*_), and following pairs (*A*-*D*, *A*-*N*_*m*_, and *A*-*r*_*c*_).

Parameters	*F*	*F*_*t*_	*P*
*A*	558.55	28.2	<0.0001
*D*	2407.39		<0.0001
*N*_*m*_	132.47		<0.001
*r*_*c*_	9298.72		<0.0001

*A*-*D*	11.32	3.450	<0.002
*A*-*N*_*m*_	16.52		<0.0001
*A*-*r*_*c*_	14.28		<0.0001

*F*-values are the Fisher statistic and *P* values are the level of significance.

**Table 5:** The values of coefficients for the exponential fit (3) of the neuron area (*A*), fractal dimension (*D*), maximum number of intersections (*N*_*m*_), and critical radius (*r*_*c*_) for the six gestational times.

Coefficients	*A* (mm^2^)	*D*	*N*_*m*_	*r*_*c*_ (*μ*m)
*a*_3_	−0.09	−1.61	−73.57	−95.57
*b*_3_	0.13	0.11	0.10	0.12
*c*_3_	0.02	1.37	31.00	40.87
